# Bacterial species and antimicrobial resistance differ between catheter and non–catheter-associated urinary tract infections: Data from a national surveillance network

**DOI:** 10.1017/ash.2022.340

**Published:** 2023-03-20

**Authors:** Stéphanie D’Incau, Andrew Atkinson, Lorenz Leitner, Andreas Kronenberg, Thomas M. Kessler, Jonas Marschall

**Affiliations:** 1 Infectious Diseases, Cantonal Hospital of Lucerne, Lucerne, Switzerland; 2 Department of Infectious Diseases, Inselspital, Bern University Hospital, Bern, Switzerland; 3 Department of Neuro-Urology, Balgrist University Hospital, University of Zürich, Zürich, Switzerland; 4 Institute for Infectious Diseases, University of Bern, Bern, Switzerland; 5 Division of Infectious Diseases, Department of Medicine, Washington University School of Medicine, St Louis, Missouri, United States

## Abstract

**Objective:**

To investigate clinically relevant microbiological characteristics of uropathogens and to compare patients with catheter-associated urinary tract infections (CAUTIs) to those with non-CAUTIs.

**Methods:**

All urine cultures from the calendar year 2019 of the Swiss Centre for Antibiotic Resistance database were analyzed. Group differences in the proportions of bacterial species and antibiotic-resistant isolates from CAUTI and non-CAUTI samples were investigated.

**Results:**

Data from 27,158 urine cultures met the inclusion criteria. *Escherichia coli*, *Klebsiella pneumoniae*, *Pseudomonas aeruginosa*, and *Proteus mirabilis* together represented 70% and 85% of pathogens identified in CAUTI and non-CAUTI samples, respectively. *Pseudomonas aeruginosa* was significantly more often detected in CAUTI samples. The overall resistance rate for the empirically often-prescribed antibiotics ciprofloxacin (CIP), norfloxacin (NOR), and trimethoprim-sulfamethoxazole (TMP-SMX) was between 13% and 31%. Except for nitrofurantoin, *E. coli* from CAUTI samples were more often resistant (*P* ≤ .048) to all classes of antibiotics analyzed, including third-generation cephalosporines used as surrogate for extended-spectrum β-lactamase (ESBL). Significanty higher resistance proportions in CAUTI samples versus non-CAUTI samples were observed for CIP (*P* = .001) and NOR (*P* = .033) in *K. pneumoniae*, for NOR (*P* = .011) in *P. mirabilis*, and for cefepime (*P* = .015), and piperacillin-tazobactam (*P* = .043) in *P. aeruginosa.*

**Conclusion:**

CAUTI pathogens were more often resistant to recommended empirical antibiotics than non-CAUTI pathogens. This finding emphasizes the need for urine sampling for culturing before initiating therapy for CAUTI and the importance of considering therapeutic alternatives.

Urinary tract infections (UTIs) represent 12%–50% of all hospital-acquired infections, with most cases being catheter-associated infections (CAUTIs).^1–3^ *Escherichia coli* is the pathogen most commonly involved in UTIs.^3,4^ CAUTIs are usually considered “complicated” UTIs and are often empirically treated with fluroquinolones. However, over the past 20 years, an increases in quinolone-resistant *E. coli* have been observed in many countries. Gram-negative bacteria producing extended-spectrum β-lactamases (ESBLs) are also on the rise.^5–8^ These shifts are making it difficult for clinicians to choose the optimal empiric therapy. Accordingly, it is critically important to understand the current situation of antimicrobial resistance (AMR) related to CAUTIs. To the best of our knowledge, no recent work from industrialized countries has specifically studied CAUTIs. A meta-analysis focused on CAUTIs in ICUs showed that these infections were mainly caused by gram-negative bacteria resistant to common antibiotics.^9^ In that study, however, CAUTIs were not compared to non-CAUTIs. Also, intensive care unit (ICU) patients represent a very specific population, and these findings may not apply to CAUTIs in general, regardless of the setting. A multicentric study conducted in 20 hospitals from 8 countries in southern Europe, as well as Turkey and Israel (where the rates of multidrug-resistant (MDR) pathogens are high) compared CAUTI rates to rates of other complicated UTIs (cUTIs).^10^ There, CAUTIs were more frequently associated with MDR gram-negative bacteria (35.2% vs 23%; *P* < .001) compared to other cUTIs, but only hospitalized patients were included. In addition, these countries are well known for high rates of antimicrobial resistance. Moreover, these researchers presented no detailed information regarding specific antibiotic susceptibilities. In another study that focused on nosocomial infections exclusively analyzed CAUTIs as a subgroup, outpatients were not included and CAUTI cases were not compared to non-CAUTI cases.^11^

As of December 2021, we were unable to find published studies directly comparing CAUTI versus non-CAUTI with a focus on AMR. Our goal was to clarify whether uropathogens and AMR characteristics vary depending on catheter association, which could provide a reference for clinicians regarding empirical antimicrobial therapy. Because the data had been anonymized, no institutional review board approval was required for conducting this analysis.

## Materials and methods

### Setting

For this analysis, we used an ANRESIS (Swiss Centre for Antibiotic Resistance, www.anresis.ch) data set containing all urine cultures, including antibiotic susceptibility testing and demographic data from the calendar year 2019. ANRESIS is a representative nationwide surveillance system, funded by the Federal Office of Public Health of Switzerland and the University of Bern that serves as a research instrument for exploring antibiotic resistance and consumption. ANRESIS collects anonymized routine microbiological data for inpatients and outpatients from microbiology laboratories, distributed homogeneously across Switzerland. We included data collected by the 32 laboratories that provided data on urinary samples for the year 2019. These 32 laboratories cover ∼70% of all urine cultures processed in Swiss medical laboratories. Only the first isolate per patient was included, unless the same patient had multiple isolates with either different pathogens or the same pathogen, but different resistance patterns. Mono- and polymicrobial urine cultures were included.

### Microbiological analyses

Antimicrobial susceptibility testing was performed according to EUCAST or CLSI guidelines at the local laboratories.^12^ All participating laboratories are approved and participate regularly in external quality programs. Isolates with intermediate susceptibility were considered resistant. Resistance to at least 1 third- or higher-generation cephalosporin was analyzed as one AMR group subsequently labeled C3/4G. If intermediate or full resistance to any C3/4G was detected, the entire group was considered resistant. This approach was used as a surrogate for ESBL production. For the AMR analysis, we focused on the commonly utilized antibiotics for the most common uropathogens. For *Escherichia coli*, *Klebsiella pneumoniae*, and *Proteus mirabilis*, these antibiotics are C3/4G, trimethoprim-sulfamethoxazole (TMP-SMX), ciprofloxacin (CIP), norfloxacin (NOR), nitrofurantoin (NIT), fosfomycin (FFM) and amoxicillin/clavulanic acid (AMC). For *Pseudomonas aeruginosa*, these antibiotics are cefepime (CEF), ceftazidime (CAZ), piperacillin-tazobactam (PIP), imipenem (IMP) and CIP.

### Variables collected and variable classification

We considered all these samples to be associated with a UTI and used the CAUTI acronym to refer to cultures from catheter-associated samples (labelled “urine catheter”). The following collection categories were included in the non-CAUTI group: “urine midstream,” “urine punction,” “urine single catheterization,” and “urine after prostatic massage.”

For analysis of distribution, we compared nosocomial versus community acquisition and types of healthcare centers (primary, secondary, or tertiary care). Only CAUTI samples were included. When available, we used the hospital entry date and sample date to extrapolate whether the infection was nosocomial (>2 days after admission) or community acquired. To distinguish geographical differences, samples were divided according to language area (ie, French-speaking Switzerland vs German-speaking Switzerland). Because the Ticino location did not record any data on CAUTI, Italian-speaking Switzerland was excluded from these analyses. Patients with samples labelled “transfer from abroad” were also excluded from analysis. Finally, all samples with missing data were excluded.

### Statistical analysis

Group differences in the proportions of bacterial species and antibiotic-resistant isolates from CAUTI and non-CAUTI samples were investigated using the χ^2^ test (or variants thereof). A 2-sided *P* value < .05 was considered statistically significant. All data were analyzed using R version 1.3.1073 software (R Foundation for Statistical Computing, Vienna, Austria).

## Results

### Urine samples

The ANRESIS database included 1,048,575 microorganism-antibiotic resistance combinations for 59,869 urine samples from 45,510 patients for the calendar year 2019. Of these 59,869 urine samples, 35,428 (59%) were of unknown origin (or information about origin was missing), 19,933 (33%) were not associated with a urinary catheter, and 4,508 (8%) were associated with a urinary catheter (Supplementary Fig. S1). Characteristics of samples according to origin are described in Table 1.

**Table 1.** Origin of Urine Culture Samples: Sociodemographic and Geographic Characteristics

Characteristic	CAUTI	Non-CAUTI	*P* Value
No.	4,508	19,933	
Age, median y (IQR)	75 (60–80)	65 (40–75)	<.001
**Sex (%)**			<.001
Female	1,919 (42.6)	13,675 (68.6)	
Male	2,587 (57.4)	6,234 (31.3)	
Unknown	2 (0.0)	24 (0.1)	
**Origin (%)**			<.001
Community-acquired	1,452 (32.2)	7,785 (39.1)	
Nosocomial	1,225 (27.2)	4,268 (21.4)	
Not defined	1,831 (40.6)	7,880 (39.5)	
**Region (%)**			
Foreign countries	65 (1.4)	295 (1.5)	
Switzerland Central East	318 (7.1)	2,053 (10.3)	
Switzerland Central West	1,295 (28.7)	3,892 (19.5)	
Switzerland East	410 (9.1)	1,556 (7.8)	
Switzerland Geneva area	0 (0.0)	47 (0.2)	
Switzerland Northast	1,332 (29.5)	6,582 (33.0)	
Switzerland Northwest	304 (6.7)	1,261 (6.3)	
Switzerland South	0 (0.0)	0 (0.0)	
Switzerland West	784 (17.4)	4,247 (21.3)	
**Hospital (%)**			
Primary	2,374 (52.7)	7,321 (36.7)	
Secondary	1,092 (24.2)	5,990 (30.1)	
Tertiary	1,042 (23.1)	6,622 (33.2)	

Note: CAUTI, catheter-associated urinary tract infection.

### Missing information and samples of unknown origin

Most of the samples in the database lacked the detailed information regarding origin (CAUTI vs non-CAUTI) and were therefore excluded from further analyses comparing CAUTI and non-CAUTI samples. Notably, some geographical areas almost exclusively registered samples of unknown origin or with missing origin (eg, Geneva and southern Switzerland); therefore, further analyses of CAUTI included none or very few samples from these areas (Table 1).

### Number of pathogens per sample

We used >2 pathogens as a surrogate for contamination. For non-CAUTI cultures, 18,332 samples (92%) had only 1 pathogen identified, 1,468 samples (7.4 %) had 2 pathogens identified, and 113 samples (0.6%) had >2 pathogens identified. For CAUTI cultures, 3,946 samples (87.3%) had 1 pathogen identified, 509 samples (11.2%) had 2 pathogens identified, and 62 samples (1.4%) had >2 pathogens identified.

### Length of stay: Community acquisition versus nosocomial infection

In both CAUTI and non-CAUTI groups, most samples lacked information needed to estimate whether the infection was community acquired or nosocomial. When the information was available, most of infections were community acquired in both groups, with a slightly higher proportion of community-acquired non-CAUTIs compared with CAUTIs (Table 1).

### Pathogen distribution in CAUTI vs non-CAUTI

*Escherichia coli* was the most frequent pathogen identified in both CAUTI and non-CAUTI samples (Table 2). The 5 most frequently identified pathogens were the same in CAUTIs and non-CAUTIs: *E. coli, K. pneumoniae, P. aeruginosa, P. mirabilis,* and *E. faecalis*. This group represented 70.2% and 81% of all pathogens identified in CAUTI and non-CAUTI samples, respectively (Table 2). We restricted AMR analyses on these pathogens, except for *E. faecalis*, which we excluded from further analyses due to its uncertain pathogenicity and its general susceptibility to penicillin or penicillin derivatives.^13^

**Table 2.** Identified Bacteria Species in CAUTI Versus Non-CAUTI Samples

Species	CAUTI	Non-CAUTI	*P* Value
No.	5,317	21,841	
Bacteria, no. (%)			<.001
*Escherichia coli*	1,694 (31.9)	11,960 (54.8)	<.001
*Enterococcus faecalis*	632 (11.9)	1,972 (9.0)	<.001
*Klebsiella pneumoniae*	591 (11.1)	2,162 (9.9)	.008
*Pseudomonas aeruginosa*	535 (10.1)	711 (3.3)	<.001
*Proteus mirabilis*	283 (5.3)	870 (4.0)	<.001
*Citrobacter* spp	198 (3.7)	559 (2.6)	<.001
*Enterobacter* spp	183 (3.4)	453 (2.1)	<.001
*Klebsiella oxytoca*	146 (2.7)	413 (1.9)	<.001

Note: CAUTI, catheter-associated urinary tract infection.

### Resistance patterns in CAUTI versus non-CAUTI pathogens

**Fig. 1.**
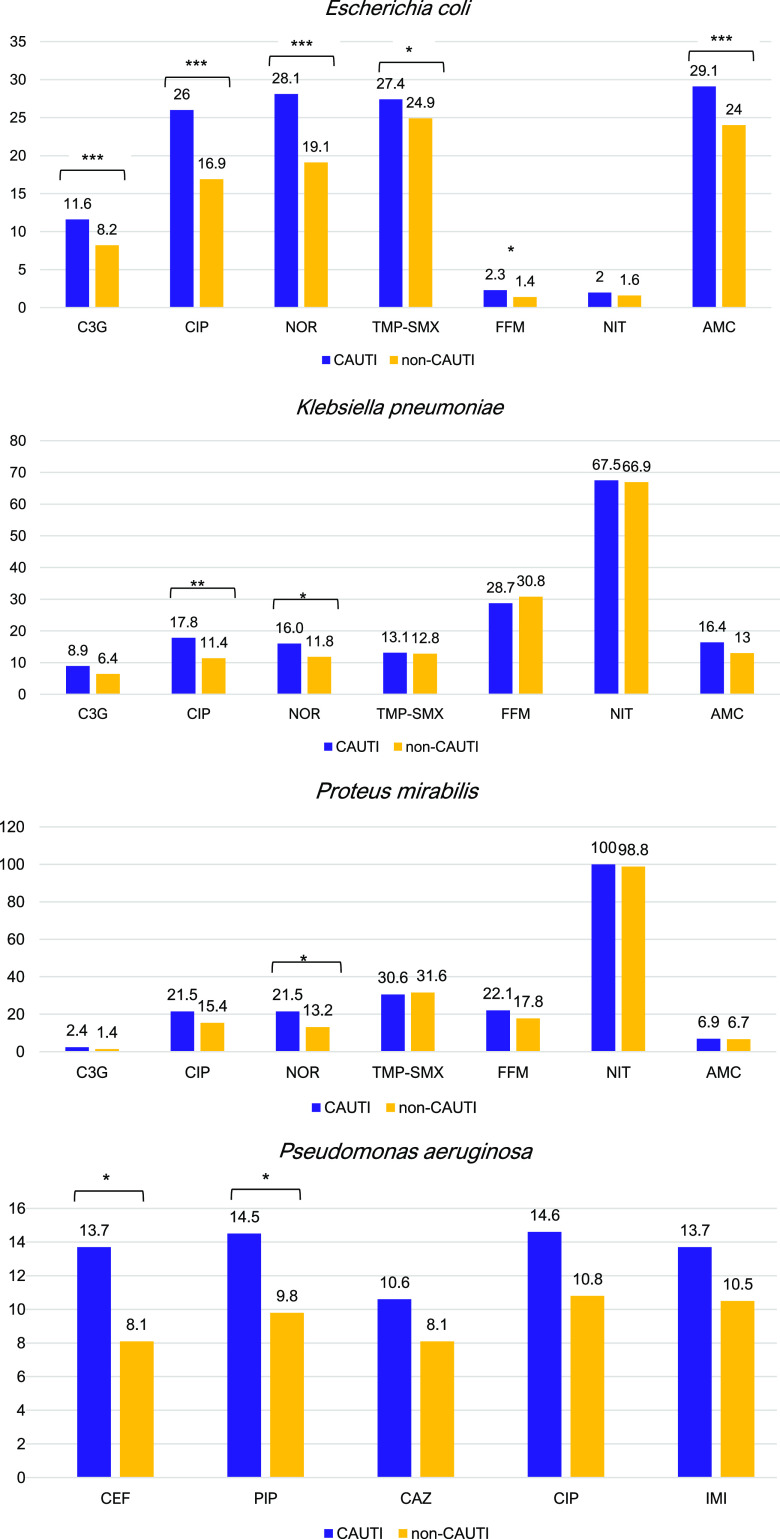
Resistance rates (%) among the most frequent uropathogens (y-axis). Note. * *P* < .05; ** *P* < .01; and *** *P* < .001. C3G, ceftriaxone; CIP, ciprofloxacin; NOR, norfloxacin; TMP-SMZ, trimethoprim-sulfamethoxazole; FFM, fosfomycin; NIT, nitrofurantoin; AMC, amoxicillin/clavulanic acid; CEF, cefepime; PIP, piperacillin-tazobactam; CAZ, ceftazidime; IMI, imipenem.

Figure 1 shows the resistance patterns comparing CAUTI and non-CAUTI samples. Except for NIT, *E. coli* from CAUTI samples were more often resistant to the analyzed classes of antibiotics than the *E. coli* from non-CAUTI samples. The difference in resistance was significant (*P* < .001) for C3/4G (ie, the surrogate for EBSL; 11.6% of CAUTI samples were resistant vs 8.2% of non-CAUTI samples), CIP (26% of CAUTI samples were resistant vs 16.9% of non-CAUTI samples), NOR (28.1% of CAUTI samples were resistant vs 19.1% of non-CAUTI samples) and AMC (29.1% of CAUTI samples were resistant vs 24% non-CAUTI samples). TMP-SMX and FFM showed significant higher resistance rates in CAUTI samples (*P* < .05), too.

For *K. pneumoniae*, the difference in resistance between CAUTI and non-CAUTI samples was statistically significant for CIP (17.8% vs 11.4% resistant, respectively; *P* = .001), and we detected a marginal difference in resistance, respectively, for NOR (16% vs 11.8%, resistant, respectively; *P* < .05). For *P. mirabilis*, the difference in resistance between CAUTI and non-CAUTI samples was significant only for NOR (21.5% vs 13.2% resistant, respectively; *P* < .05). For *P. aeruginosa*, the differences in resistance between CAUTI and non-CAUTI samples were significant for CEF (13.7% vs 8.1% resistant, respectively; *P* < .05) and PIP (14.5% vs 9.8% resistant, respectively; *P* < .05). Detailed resistance data are shown in Supplementary Table S1.

### Further analyses of CAUTI samples

*Hospital category.* Overall, 52.7% of all CAUTI samples were obtained from primary care centers, 24.2% were obtained from secondary care centers, and 23.1% were obtained from tertiary medical centers. The distributions of pathogens were similar within the 3 types of centers, and *E. coli* was the most commonly identified bacteria. *Escherichia coli* specimens from tertiary-care centers were significantly more often resistant to C3/4G, AMC, and TMP-SMX. *K. pneumoniae* infections in tertiary-care centers were more often resistant to CIP. Samples from secondary- and tertiary-care centers showed a higher resistance to FFM compared to primary care centers. *P. mirabilis* from tertiary care centers were significantly more often resistant to C3/4G. There was no significant difference in resistance profiles from *P. aeruginosa* between different types of centers (Supplementary Table S2).

*Geographical area. Escherichia coli* was the most frequent pathogen in all regions (Supplementary Table S3). There was no significant difference in the resistance profile of *E. coli*, *P. mirabilis*, and *P. aeruginosa* across regions. For *K. pneumoniae*, resistance to CIP was higher in samples from French-speaking regions compared to German-speaking regions. There was no significant difference in C3/4G resistance between regions (Supplementary Table S4).

*Nosocomial versus community-acquired CAUTI*. In the 2,677 samples evaluated, there was no difference in AMR between community-acquired and nosocomial CAUTI pathogens (Supplementary Table S5).

## Discussion

### Summary of the principal findings

In this study, we evaluated resistance patterns in a large group of urine samples collected in 2019. We specifically focused on comparing CAUTI versus non-CAUTI samples. To the best of our knowledge, this direct comparison has not been studied before. In terms of pathogens, the most relevant difference concerned *P. aeruginosa*, which represented 10% of CAUTI pathogens versus only 3.3% of non-CAUTIs. Even though the other pathogens were distributed similarly between CAUTIs and non-CAUTIs, their resistance profiles differed significantly. The main difference in terms of resistance was observed for *E. coli*, with a higher resistance rate to all antibiotics except for nitrofurantoin. *K. pneumoniae* und *P. mirabilis* showed differences only for resistance to quinolones. We did not find any significant difference in AMR profiles within the CAUTI group when nosocomial and community-acquired infections among CAUTI patients were compared (although missing data on origin considerably reduced the sample size).

### Strengths

The biggest strength of our study was the large data set. We analyzed data from 32 laboratories covering ∼70% of all urine cultures processed in Swiss medical laboratories during the study year. With 24,441 samples, we believe our findings are a solid representation of the current ecology of urinary pathogens in Switzerland.

### Limitations

Our study had several limitations. A main concern was the missing data describing the type of sample (ie, whether it was catheter related or not) that led us to exclude many samples from the analysis. We investigated the excluded data as a separate group, and the data were similar in terms of pathogen distribution (data not shown). Therefore, had these data been included, these data may not have affected our conclusions to a great extent.

We did not have clinical information for the patients from whom the urine samples were taken. Because urine should not be sampled in the absence of suspected infection, it is very likely that most of these results were associated with the prescription of an antibiotic. However, we were unable to discern between bacteriuria und infection given the absence of clinical information. Nevertheless, this distinction may not be of great relevance in a resistance prevalence study because colonizers reflect the resistance situation and can later become true pathogens of infections.

From a microbiological point of view, we did not examine the prevalence of overlapping resistance (eg, multiresistant gram-negative bacilli) and no data were available from the participating laboratories regarding an ESBL confirmation test (we used C3/4G resistance as a surrogate instead).

As the Geneva area and Ticino (ie, the Italian-speaking area) provided information regarding origin only for very few samples captured by ANRESIS, CAUTI cultures from these regions could not be analyzed. The geographical proximity and societal links with Italy for Ticino and France for Geneva may mean that these regions could present differently in terms of the resistance patterns.

### Implications for practice or policy

CAUTIs are more often associated with resistant bacteria. Whenever a catheter (ie, the apparent risk factor for acquiring more resistant pathogens) can be removed, it should be done. A urine culture before initiating antibiotics is essential and the therapy should subsequently be adapted. CAUTIs are considered a complicated UTI; therefore, empirical therapy often consists either of ceftriaxone for inpatients or ciprofloxacin for outpatients. However, current guidelines recommend using local antimicrobial resistance to help guide empirical treatment.^14,15^ Our data on local antimicrobial resistance shows that in this group of complicated UTIs, ciprofloxacin resistance reached 14.6%–26% for the 4 main pathogens. For *E. coli* and *K. pneumoniae*, ceftriaxone resistance rates were 11.6% and 8.9%, respectively, and ceftriaxone would intrinsically not be active in case of *P. aeruginosa* infections. Prescribers should therefore be alert that an adaptation of therapy will often be necessary for inpatients and outpatients upon receipt of the antimicrobial susceptibilities. In the case of septic shock due to CAUTI, empirical therapy for ESBL-producing pathogens and *Pseudomonas* would be sensible. From a surveillance point of view sample locations should be indicated more accurately in future to make sure analyses are reliable.

### Implications for future research

All oral agents encountered in this study presented a relatively high resistance rate (ie, exceeding 10%) except for fosfomycin and nitrofurantoin in the case of *E. coli* infections. These agents are often not recommended in cases of complicated UTI^15^ and are probably underutilized in CAUTI cases. They are also less often associated with collateral damage to the host. In the light of these findings, their role for CAUTI treatment should be re-evaluated.

## Supplementary material

For supplementary material accompanying this paper visit http://doi.org/10.1017/ash.2022.340. click here to view supplementary material
