# Supplementary material for: Bacterial species and antimicrobial resistance differ between catheter and non–catheter-associated urinary tract infections: Data from a national surveillance network
Source: Antimicrob Steward Healthc Epidemiol. 2023 Mar 20;3(1):e55. doi: 10.1017/ash.2022.340 (PMC10031580; doi:10.1017/ash.2022.340)
Supplement: Supplementary file 1 [file S2732494X22003400sup001.docx]

Figure S1. Flowchart of urine culture isolates included in the study.


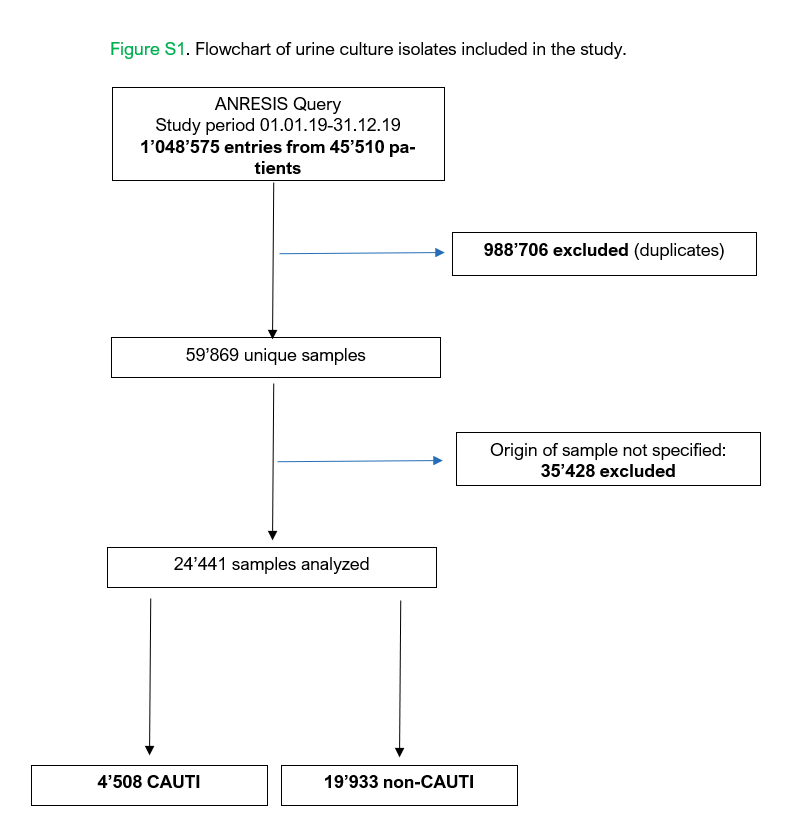


Table S1 Resistance Pattern, CAUTI vs non-CAUTI

|  |  | ***E. coli*** | | | ***K. pneumoniae*** | | | ***P. mirabilis*** | | | ***P. aeruginosa*** | | |
| --- | --- | --- | --- | --- | --- | --- | --- | --- | --- | --- | --- | --- | --- |
|  |  | CAUTI | non-CAUTI | p-value | CAUTI | non-CAUTI | p-value | CAUTI | non-CAUTI | p-value | CAUTI | non-CAUTI | p-value |
| C3G (%) | n | 1316 | 9678 |  | 436 | 1601 |  | 208 | 663 |  |  |  |  |
|  | R | 153 (11.6) | 789 (8.2) | **<0.001** | 39 (8.9) | 103 (6.4) | 0.086 | 5 (2.4) | 9 (1.4) | 0.465 |  |  |  |
| CIP (%) | n | 1183 | 9075 |  | 370 | 1399 |  | 172 | 599 |  | 350 | 528 |  |
|  | R | 307 (26.0) | 1533 (16.9) | **<0.001** | 66 (17.8) | 160 (11.4) | **0.001** | 37 (21.5) | 92 (15.4) | 0.074 | 51 (14.6) | 57 (10.8) | 0.118 |
| NOR (%) | n | 1257 | 7369 |  | 399 | 1264 |  | 186 | 470 |  |  |  |  |
|  | R | 353 (28.1) | 140 (19.1) | **<0.001** | 64 (16.0) | 149 (11.8) | **0.033** | 40 (21.5) | 62 (13.2) | **0.011** |  |  |  |
| TMP-SMX (%) | n | 1423 | 10500 |  | 451 | 1675 |  | 219 | 699 |  |  |  |  |
|  | R | 390 (27.4) | 2619 (24.9) | **0.048** | 59 (13.1) | 215 (12.8) | 0.953 | 67 (30.6) | 221 (31.6) | 0.841 |  |  |  |
| FFM (%) | n | 1405 | 10444 |  | 341 | 1291 |  | 154 | 494 |  |  |  |  |
|  | R | 32 (2.3) | 148 (1.4) | **0.018** | 98 (28.7) | 398 (30.8) | 0.496 | 34 (22.1) | 88 (17.8) | 0.287 |  |  |  |
| AMC (%) | n | 1403 | 10790 |  | 458 | 1708 |  | 216 | 690 |  |  |  |  |
|  | R | 408 (29.1) | 2586 (24.0) | **<0.001** | 75 (16.4) | 222 (13.0) | 0.073 | 15 (6.9) | 46 (6.7) | 1 |  |  |  |
| NIT (%) | n | 1276 | 9641 |  | 305 | 1077 |  | 159 | 518 |  |  |  |  |
|  | R | 25 (2.0) | 154 (1.6) | 0.401 | 206 (67.5) | 720 (66.9) | 0.875 | 159 (100.0) | 512 (98.8) | 0.379 |  |  |  |
| CEF (%) | n |  |  |  |  |  |  |  |  |  | 329 | 481 |  |
|  | R |  |  |  |  |  |  |  |  |  | 45 (13.7) | 39 (8.1) | **0.015** |
| PIP (%) | n |  |  |  |  |  |  |  |  |  | 344 | 521 |  |
|  | R |  |  |  |  |  |  |  |  |  | 50 (14.5) | 51 (9.8) | **0.043** |
| CAZ (%) | n |  |  |  |  |  |  |  |  |  | 369 | 507 |  |
|  | R |  |  |  |  |  |  |  |  |  | 39 (10.6) | 41 (8.1) | 0.254 |
| IMI (%) | n |  |  |  |  |  |  |  |  |  | 313 | 495 |  |
|  | R |  |  |  |  |  |  |  |  |  | 43 (13.7) | 52 (10.5) | 0.201 |

*C3G = Ceftriaxone; CIP = Ciprofloxacin; NOR = Norfloxacin; TMP-SMZ = trimethoprim-sulfamethoxazole; FFM = fosfomycin; NIT = Nitrofurantoin; AMC = Amoxicillin/clavulanic acid; CEF = cefepime; PIP = piperacillin-tazobactam; CAZ = ceftazidime; IMI = Imipenem*

Table S2 Resistance profile, CAUTI samples, Hospital category

1. *Escherichia coli*

|  |  | **Primary** | **Secondary** | **Tertiary** | **P value** |
| --- | --- | --- | --- | --- | --- |
| **C3G (%)** | n | 709 | 374 | 233 |  |
|  | R | 79 (11.1) | 34 (9.1) | 40 (17.2) | **0.009** |
| **CIP (%)** | n | 647 | 333 | 203 |  |
|  | R | 160 (24.7) | 81 (24.3) | 66 (32.5) | 0.064 |
| **NOR (%)** | n | 712 | 303 | 237 |  |
|  | R | 207 (28.9) | 83 (27.4) | 63 (26.6) | 0.757 |
| **TMP-SMX (%)** | n | 712 | 405 | 306 |  |
|  | R | 181 (25.4) | 108 (26.7) | 101 (33.0) | **0.042** |
| **FFM (%)** | n | 685 | 412 | 308 |  |
|  | R | 16 (2.3) | 9 (2.2) | 7 (2.3) | 0.987 |
| **NIT (%)** | n | 679 | 392 | 205 |  |
|  | R | 14 (2.1) | 4 (1.0) | 7 (3.4) | 0.129 |
| **AMC (%)** | n | 707 | 384 | 312 |  |
|  | R | 166 (23.5) | 121 (31.5) | 121 (38.8) | **<0.001** |

1. *Klebsiella pneumonia*

|  |  | **Primary** | **Secondary** | **Tertiary** | **P value** |
| --- | --- | --- | --- | --- | --- |
| **C3G (%)** | n | 213 | 139 | 84 |  |
|  | R | 16 (7.5) | 10 (7.2) | 13 (15.5) | 0.065 |
| **CIP (%)** | n | 187 | 113 | 70 |  |
|  | R | 29 (15.5) | 17 (15.0) | 20 (28.6) | **0.033** |
| **NOR (%)** | n | 208 | 100 | 91 |  |
|  | R | 37 (17.8) | 15 (15.0) | 12 (13.2) | 0.576 |
| **TMP-SMX (%)** | n | 199 | 137 | 115 |  |
|  | R | 20 (10.1) | 17 (12.4) | 22 (19.1) | 0.068 |
| **FFM (%)** | n | 153 | 113 | 75 |  |
|  | R | 30 (19.6) | 45 (39.8) | 23 (30.7) | **0.001** |
| **NIT (%)** | n | 164 | 85 | 56 |  |
|  | R | 115 (70.1) | 54 (63.5) | 37 (66.1) | 0.555 |
| **AMC (%)** | n | 200 | 138 | 120 |  |
|  | R | 29 (14.5) | 23 (16.7) | 23 (19.2 | 0.547 |

1. *Proteus mirabilis*

|  |  | **Primary** | **Secondary** | **Tertiary** | **P value** |
| --- | --- | --- | --- | --- | --- |
| **C3G (%)** | n | 96 | 65 | 47 |  |
|  | R | 1 (1.0) | 0 (0.0) | 4 (8.5) | **0.007** |
| **CIP (%)** | n | 84 | 49 | 39 |  |
|  | R | 21 (25.0) | 8 (16.3) | 8 (20.5) | 0.494 |
| **NOR (%)** | n | 99 | 44 | 43 |  |
|  | R | 25 (25.3) | 7 (15.9) | 8 (18.6) | 0.396 |
| **TMP-SMX (%)** | n | 97 | 63 | 59 |  |
|  | R | 27 (27.8) | 18 (28.6) | 22 (37.3) | 0.424 |
| **FFM (%)** | n | 70 | 46 | 38 |  |
|  | R | 20 (28.6) | 9 (19.6) | 5 (13.2) | 0.162 |
| **NIT (%)** | n | 98 | 38 | 23 |  |
|  | R | 98 (100.0) | 38 (100.0) | 23 (100.0) | NA |
| **AMC (%)** | n | 95 | 61 | 60 |  |
|  | R | 7 (7.4) | 3 (4.9) | 5 (8.3) | 0.743 |

1. *Pseudomonas aeruginosa*

|  |  | **Primary** | **Secondary** | **Tertiary** | **P value** |
| --- | --- | --- | --- | --- | --- |
| **CEF (%)** | n | 142 | 100 | 87 |  |
|  | R | 21 (14.8) | 17 (17.0) | 7 (8.0) | 0.181 |
| **CIP (%)** | n | 148 | 123 | 79 |  |
|  | R | 26 (17.6) | 17 (13.8) | 8 (10.1) | 0.305 |
| **PIP (%)** | n | 126 | 130 | 88 |  |
|  | R | 21 (16.7) | 20 (15.4) | 9 (10.2) | 0.396 |
| **CAZ (%)** | n | 152 | 129 | 88 |  |
|  | R | 18 (11.8) | 14 (10.9) | 7 (8.0) | 0.635 |
| **IMI (%)** | n | 139 | 90 | 84 |  |
|  | R | 20 (14.4) | 13 (14.4) | 10 (11.9) | 0.85 |

*C3G = Ceftriaxone; CIP = Ciprofloxacin; NOR = Norfloxacin; TMP-SMZ = trimethoprim-sulfamethoxazole; FFM = fosfomycin; NIT = Nitrofurantoin; AMC = Amoxicillin/clavulanic acid; CEF = cefepime; PIP = piperacillin-tazobactam; CAZ = ceftazidime; IMI = Imipenem*

Table S3 Pathogen Distribution: comparison between geographical regions

|  | **German speaking** | **French speaking** |
| --- | --- | --- |
| **n** | **4310** | **881** |
| **Species (%)** |  |  |
| *E.coli* | 1334 (31.0) | 323 (36.7) |
| Others | 595 (13.8) | 116 (13.2) |
| *E.faecalis* | 491 (11.4) | 119 (13.5) |
| *P.aeruginosa* | 453 (10.5) | 66 (7.5) |
| *P.mirabilis* | 225 (5.2) | 52 (5.9) |
| *Enterobacter spp.* | 154 (3.6) | 25 (2.8) |
| *E.faecium* | 80 (1.9) | 35 (4.0) |
| *Enterococcus spp*.  (not identified to species level) | 116 (2.7) | 0 (0.0) |
| *K.oxytoca* | 131 (3.0) | 13 (1.5) |
| not specified | 69 (1.6) | 27 (3.1) |
|  |  |  |

Table S4 Resistance profile, CAUTI samples, Region

1. *Escherichia coli*

|  |  | **German speaking** | **French speaking** | **P value** |
| --- | --- | --- | --- | --- |
| **C3G (%)** | n | 1020 | 271 |  |
|  | R | 124 (12.2) | 25 (9.2) | 0.217 |
| **CIP (%)** | n | 916 | 247 |  |
|  | R | 250 (27.3) | 52 (21.1) | 0.057 |
| **NOR (%)** | n | 354 | 37 |  |
|  | R | 52 (14.7) | 9 (24.3) | 0.194 |
| **TMP-SMX (%)** | n | 1145 | 255 |  |
|  | R | 309 (27.0) | 74 (29.0) | 0.561 |
| **FFM (%)** | n | 1160 | 224 |  |
|  | R | 23 (2.0) | 8 (3.6) | 0.221 |
| **NIT (%)** | n | 1014 | 255 |  |
|  | R | 19 (1.9) | 6 (2.4) | 0.81 |
| **AMC (%)** | n | 1133 | 247 |  |
|  | R | 350 (30.9) | 49 (19.8) | **0.001** |

1. *Klebsiella pneumoniae*

|  |  | **German speaking** | **French speaking** | **P value** |
| --- | --- | --- | --- | --- |
| **C3G (%)** | n | 363 | 64 |  |
|  | R | 28 (7.7) | 7 (10.9) | 0.536 |
| **CIP (%)** | n | 303 | 58 |  |
|  | R | 46 (15.2) | 17 (29.3) | **0.016** |
| **NOR (%)** | n | 354 | 37 |  |
|  | R | 52 (14.7) | 9 (24.3) | 0.194 |
| **TMP-SMX (%)** | n | 386 | 57 |  |
|  | R | 44 (11.4) | 12 (21.1) | 0.067 |
| **FFM (%)** | n | 298 | 39 |  |
|  | R | 89 (29.9) | 9 (23.1) | 0.490 |
| **NIT (%)** | n | 279 | 24 |  |
|  | R | 187 (67.0) | 19 (79.2) | 0.320 |
| **AMC (%)** | n | 389 | 61 |  |
|  | R | 60 (15.4) | 12 (19.7) | 0.513 |

1. *Proteus mirabilis*

|  |  | **German speaking** | **French speaking** | **P value** |
| --- | --- | --- | --- | --- |
| **C3G (%)** | n | 160 | 46 |  |
|  | R | 3 (1.9) | 1 (2.2) | 1.00 |
| **CIP (%)** | n | 128 | 43 |  |
|  | R | 28 (21.9) | 9 (20.9) | 1.00 |
| **NOR (%)** | n | 156 | 29 |  |
|  | R | 34 (21.8) | 6 (20.7) | 1.00 |
| **TMP-SMX (%)** | n | 173 | 45 |  |
|  | R | 55 (31.8) | 11 (24.4) | 0.439 |
| **FFM (%)** | n | 127 | 26 |  |
|  | R | 29 (22.8) | 5 (19.2) | 0.886 |
| **NIT (%)** | n | 126 | 32 |  |
|  | R | 126 (100.0) | 32 (100.0) | NA |
| **AMC (%)** | n | 175 | 39 |  |
|  | R | 11 (6.3) | 3 (7.7) | 1.00 |

1. *Pseudomonas aeruginosa*

|  |  | **German speaking** | **French speaking** | **P value** |
| --- | --- | --- | --- | --- |
| **CEF (%)** | n | 44 | 280 |  |
|  | R | 6 (13.6) | 38 (13.6) | 1 |
| **PIP (%)** | n | 43 | 295 |  |
|  | R | 5 (11.6) | 44 (14.9) | 0.734 |
| **CAZ (%)** | n | 44 | 320 |  |
|  | R | 3 (6.8) | 35 (10.9) | 0.565 |
| **IMP (%)** | n | 42 | 266 |  |
|  | R | 12 (28.6) | 30 (11.3) | 0.005 |
| **CIP (%)** | n | 46 | 298 |  |
|  | R | 9 (19.6) | 39 (13.1) | 0.341 |

*C3G = Ceftriaxone; CIP = Ciprofloxacin; NOR = Norfloxacin; TMP-SMZ = trimethoprim-sulfamethoxazole; FFM = fosfomycin; NIT = Nitrofurantoin; AMC = Amoxicillin/clavulanic acid; CEF = cefepime; PIP = piperacillin-tazobactam; CAZ = ceftazidime; IMI = Imipenem*

Table S5 Resistance profile, CAUTI samples, nosocomial vs community-acquired (CA)

1. *Escherichia coli*

|  |  | **CA** | **Nosocomial** | **P value** |
| --- | --- | --- | --- | --- |
| **C3G** | n | 446 | 346 |  |
|  | r | 45 (10.1) | 43 (12.4) | 0.355 |
| **CIP** | n | 397 | 322 |  |
|  | r | 99 (24.9) | 80 (24.8) | 1 |
| **FFM** | n | 502 | 378 |  |
|  | r | 140 (27.9) | 102 (27.0) | 0.825 |
| **NIT** | n | 501 | 370 |  |
|  | r | 11 (2.2) | 6 (1.6) | 0.721 |
| **NOR** | n | 435 | 281 |  |
|  | r | 114 (26.2) | 79 (28.1) | 0.635 |
| **TMP-SMX** | n | 502 | 378 |  |
|  | r | 140 (27.9) | 102 (27.0) | 0.825 |
| **AMC** | n | 482 | 364 |  |
|  | r | 129 (26.8) | 101 (27.7) | 0.81 |

1. *Klebsiella pneumoniae*

|  |  | **CA** | **Nosocomial** | **P value** |
| --- | --- | --- | --- | --- |
| **C3G** | n | 147 | 120 |  |
|  | r | 11 (7.5) | 8 (6.7) | 0.985 |
| **CIP** | n | 116 | 103 |  |
|  | r | 14 (12.1) | 15 (14.6) | 0.731 |
| **FFM** | n | 162 | 117 |  |
|  | r | 15 (9.3) | 12 (10.3) | 0.942 |
| **NIT** | n | 126 | 78 |  |
|  | r | 71 (56.3) | 51 (65.4) | 0.258 |
| **NOR** | n | 134 | 92 |  |
|  | r | 16 (11.9) | 8 (8.7) | 0.577 |
| **TMP-SMX** | n | 162 | 117 |  |
|  | r | 15 (9.3) | 12 (10.3) | 0.942 |
| **AMC** | n | 164 | 124 |  |
|  | r | 19 (11.6) | 21 (16.9) | 0.259 |

1. *Proteus mirabilis*

|  |  | **CA** | **Nosocomial** | **P value** |
| --- | --- | --- | --- | --- |
| **C3G** | n | 60 | 76 |  |
|  | r | 0 | 0 | NA |
| **TMP-SMX** | n | 67 | 75 |  |
|  | r | 18 (26.9) | 21 (28.0) | 1 |
| **NOR** | n | 55 | 54 |  |
|  | r | 13 (23.6) | 15 (27.8) | 0.783 |
| **NIT** | n | 50 | 55 |  |
|  | r | 50 (100.0) | 55 (100.0) | NA |
| **FFM** | n | 67 | 75 |  |
|  | r | 18 (26.9) | 21 (28.0) | 1 |
| **CIP** | n | 49 | 63 |  |
|  | r | 10 (20.4) | 18 (28.6) | 0.441 |
| **AMC** | n | 67 | 71 |  |
|  | r | 4 (6.0) | 6 (8.5) | 0.816 |

1. *Pseudomonas aeruginosa*

|  |  | **CA** | **Nosocomial** | **P value** |
| --- | --- | --- | --- | --- |
| **CIP** | n | 109 | 130 |  |
|  | r | 14 (12.8) | 15 (11.5) | 0.913 |
| **PIP** | n | 96 | 112 |  |
|  | r | 10 (10.4) | 13 (11.6) | 0.959 |
| **CEF** | n | 95 | 103 |  |
|  | r | 9 (9.5) | 13 (12.6) | 0.633 |
| **IMP** | n | 92 | 97 |  |
|  | r | 12 (13.0) | 14 (14.4) | 0.947 |
| **CAZ** | n | 108 | 126 |  |
|  | r | 7 (6.5) | 10 (7.9) | 0.861 |

*C3G = Ceftriaxone; CIP = Ciprofloxacin; NOR = Norfloxacin; TMP-SMZ = trimethoprim-sulfamethoxazole; FFM = fosfomycin; NIT = Nitrofurantoin; AMC = Amoxicillin/clavulanic acid; CEF = cefepime; PIP = piperacillin-tazobactam; CAZ = ceftazidime; IMI = Imipenem*
